# Editorial: Enteric glia in intestinal homeostasis

**DOI:** 10.3389/fimmu.2025.1576963

**Published:** 2025-03-03

**Authors:** Jay V. Patankar, Marlene M. Hao, Reiner Schneider, Fränze Progatzky

**Affiliations:** ^1^ Department of Medicine1, Universitätsklinikum Erlangen and Friedrich-Alexander-Universität Erlangen-Nuremberg (FAU), Erlangen, Germany; ^2^ Department of Anatomy and Physiology, The University of Melbourne, Parkville, VIC, Australia; ^3^ Department of Surgery, Medical Faculty, University Hospital Bonn, Bonn, Germany; ^4^ The Kennedy Institute of Rheumatology, University of Oxford, Oxford, United Kingdom

**Keywords:** enteric glia, gut motility, gut microbiota, neuroimmune interaction, enteric glia-epithelial homeostasis

The enteric nervous system (ENS) is often referred to as the “second brain” due to its complex neural networks that regulate gut function independently of the central nervous system. Within this intricate system, enteric glial cells play a crucial yet often underappreciated role in maintaining intestinal homeostasis. Named as the “glue” of the nervous system, enteric glial cells were once thought only to provide structural support. They are now recognised as dynamic regulators of gut physiology, influencing gut motility, immune responses, epithelial barrier integrity, and enteric neuronal function (1, 2). Emerging research also suggests that enteric glial dysfunction is implicated in various gastrointestinal disorders, from inflammatory bowel disease to disorders of gut motility. This Frontiers Research Topic *Enteric glia in intestinal homeostasis* introduces novel research into multiple different aspects of enteric glial function, including their role in Wnt signalling, responses to extracellular matrix substrates, and communication with the gut microbiome, highlighting the diversity of enteric glial activity.

Santosh et al. provide an up-to-date review on the current understanding of enteric glial diversity, their interaction with other gut cells and the emerging roles of enteric glia during intestinal diseases, such as infectious colitis, inflammatory bowel disease, and cancer.

Increasing mechanistic evidence is emerging that suggests that glial cells are a fundamental component of the gastrointestinal tissue circuitry, actively participating in critical signaling pathways essential for intestinal homeostasis. One example is Wnt signaling, which plays a key role in maintaining stem cell homeostasis within the intestinal crypt. Enteric glial cells have been shown to contribute to this regulation (3). However, the role of other components of the enteric nervous system in Wnt signaling remains largely uncharacterized. In this Research Topic, Scharr et al. describe the spatial gene expression profile of Wnt-signaling components in the ENS. Employing RNAscope HiPlex-assays, the authors were able to detect the tight regulation of Wnt-signaling components and compared their results with published scRNA and RiboTag-based RNA sequencing datasets. Their descriptive analysis showed that several components of the multidimensional regulatory network of the Wnt-signaling pathway are detectable in the murine ENS. Using data mining approaches, their study revealed, that several Wnt-related molecules are expressed by enteric glial cell clusters and are dynamically regulated during the gut inflammation, indicating a broad impact of the Wnt pathway in the ENS and its cellular homeostasis in health and inflammation.

In line with the growing recognition that enteric glial cells play a crucial role in maintaining the epithelial barrier, Bali and Grubišić provide a fresh perspective on mucosal enteric glial cells, highlighting their diverse roles in maintaining and restoring gut homeostasis. Integrated into the complex tissue network of the intestinal mucosa, this glial cell subpopulation is strategically positioned to interact with mucosal immune cells, intestinal epithelial cells, and both intrinsic (ENS) and extrinsic (CNS) innervation. The authors propose that under steady-state conditions, enteric glia actively regulate gut reflexes, such as secretomotor function, while their role in epithelial barrier maintenance appears redundant. However, during inflammation or injury, mucosal glia make essential contributions to tissue repair and immune interactions to restore intestinal homeostasis. The development of new transgenic tools to target specific enteric glial cell subtypes will present renewed opportunities to study distinct glial cell subtypes and offer exciting insights into their specialized functions in mucosal health and disease.

The unique extracellular matrix composition and its remodeling in pathological conditions enable enteric glia to regulate ENS homeostasis. The diverse anatomical niches in which enteric glia are operational, demand a complex glia-ECM interaction in shaping their function, a feature challenging to investigate *in vivo*. Schneider et al. investigated the impact of commonly used artificial ECM compositions on primary enteric glia, keeping in mind the growing number of studies employing primary glia cultures in *ex vivo* assays. Network formation assays, immunohistochemical analysis, and comparative transcriptomics of primary glia grown on Matrigel, laminin, poly-L-ornithine, lysines, collagens, and fibronectin identified the first two as superior over other coatings, supporting greater neuronal differentiation and cell proliferation. Furthermore, to uncover the impact of the ECM coatings on glia activation, the authors stimulated primary glia grown on the three most promising ECM coatings with recombinant IL1ß. Although, the overall release of CCL2 and IL6, post-stimulation was comparable between Matrigel, laminin, and poly-L-ornithine, the differential expression of several genes and enrichment of functional ontologies revealed coating-dependent differences. These findings highlight the importance of the ECM composition when analyzing enteric glia *ex vivo* and highlight the importance of considering glia-ECM interactions in future studies.

There has been growing interest in understanding how microbiota influence the degree of neuronal innervation and the number of mucosal glia (4, 5). In this Research Topic, Kato et al. show how constitutive TLR4 activation in glia drives the release of ciliary neurotrophic factor (CNTF), which is essential in maintaining enteric nerve innervations in apical reaches of the mucosa. A rapid increase in innervation was observed within hours in mice that received recombinant CNTF, indicative of an axon elongation mechanism akin to stimulus-mediated local translation (6). This microbiota-glia/TLR4-CNTF circuit seems to play a role in maintaining homeostatic innervation in the proximal colon. However, the mechanism behind the rapid nature of this response and conservation in human gut remains to be investigated.

Despite significant progress in understanding the roles of enteric glial cells in gut function and intestinal homeostasis, many critical questions remain unanswered. Differentiating between the multiple subtypes of enteric glia remains a challenge, and whether there is plasticity between the different glial subpopulations remains unknown (7–9). Further research exploring whether specific populations of enteric glia have distinct roles in communicating with different cellular components of the gut is needed. With advances in stem cell technology, spatial sequencing, and *in vivo* imaging, we are poised to unravel the full spectrum of enteric glial functions and their broader implications for health and disease. Bridging these knowledge gaps will not only deepen our fundamental understanding of gut physiology but may also lead to innovative treatments for many digestive diseases.
